# The impact of supplementation with calcium and naloxone to TRIS and Lepus extenders on the quality of refrigerated rabbit semen

**DOI:** 10.3389/fvets.2026.1830161

**Published:** 2026-05-28

**Authors:** Viola Zappone, Salvatore Alonge, Roberta Angelastri, Alessandro Troisi, Romina Marcoccia, Marco Quartuccio, Giulio Guido Aiudi

**Affiliations:** 1 Department of Veterinary Medicine, University of Messina, Messina, Italy; 2 Società Veterinaria “Il Melograno” Srl, Sesto Calende, Italy; 3 Department of Veterinary Medicine, University of Bari “Aldo Moro”, Bari, Italy; 4 Department of Bioscience, University of Camerino, Camerino, Italy

**Keywords:** calcium ions, cooled semen, extender, ion homeostasis, naloxone, rabbit reproduction

## Abstract

Maintaining sperm quality during storage is essential for optimizing reproductive efficiency in rabbit breeding. This study investigated the effects of modulating calcium metabolism and the endogenous opioid system on rabbit sperm function. Two commercial extenders (TRIS and Lepus) were supplemented with calcium chloride (CaCl_2_) and naloxone (Nx), either individually or in combination. Semen was collected from 20 New Zealand White (NZW) males, pooled, diluted, and assigned to eight experimental treatments. Sperm motility, kinematic parameters, vitality, and morphology were assessed after storage, and data were analyzed by two-way ANOVA. The results showed that treatment was the main factor affecting sperm quality parameters, whereas the effect of diluent was significant only for selected variables. The results revealed significant differences among experimental groups, with naloxone-treated samples (alone or combined with CaCl_2_) showing the highest sperm quality. In particular, total motility (MOT) increased from approximately 56–71% in control and CaCl_2_ groups to about 77–85% in Nx-treated groups, while progressive motility (PMOT) improved from approximately 44–57% to 73–82%. Similarly, kinetic parameters (e.g., VAP and VCL) were markedly enhanced in Nx and Nx + CaCl_2_ groups (reaching ~60–69 μm/s and ~150–159 μm/s, respectively), whereas CaCl_2_ alone consistently reduced all motility-related parameters. Sperm vitality also improved in Nx-containing treatments (up to ~0.95–0.96), compared to lower values in CaCl_2_-only groups (~0.77–0.92), while the percentage of abnormalities was reduced, particularly in TRIS-based and Nx-supplemented samples (down to ~2–4%). The combined Nx + CaCl_2_ treatments consistently showed the best overall performance across most parameters. The synergistic effect observed in the Nx + CaCl_2_ groups suggests that naloxone may regulate Ca^2+^ influx and intracellular homeostasis, preventing detrimental effects associated with calcium overload. In conclusion, pharmacological modulation of the opioid system, particularly through low concentrations of naloxone, significantly improves rabbit semen quality and, when combined with Ca^2+^, further enhances sperm functional parameters. These findings provide new perspectives for optimizing artificial insemination (AI) protocols in rabbit production systems.

## 1 Introduction

Artificial insemination (AI) is the most widely used reproductive biotechnology in farm animals and is essential for improving genetic quality and production performance (1). In rabbits, AI has quickly become standard practice in commercial production systems, particularly for high-performance breeds such as New Zealand White (NZW) and Californian rabbits. These breeds are selected for their fertility, semen quality, and ability to adapt to intensive farming conditions (2, 3). Currently, most commercial rabbit farms use fresh or chilled semen as this method ensures high sperm viability while remaining more cost-effective than cryopreservation (4–6).

Spermatozoa are highly specialized cells that have evolved to transfer the paternal genome to the oocyte. This process requires a substantial amount of energy, primarily in the form of adenosine triphosphate (ATP), which supports key functions such as motility, capacitation, and acrosome reaction. These functions are all essential for fertilization (7). Among the factors regulating these processes, calcium ions (Ca^2+^) play a central role in sperm physiology (8, 9). In human spermatozoa, low intracellular Ca^2+^ levels have been associated with reduced premature capacitation, thereby prolonging cell viability (10). Conversely, high concentrations may induce spontaneous acrosomal exocytosis and other detrimental effects (11). However, its role in mammals remains only partially understood. In mammalian spermatozoa, Ca^2+^ acts as a key regulator of multiple functional processes through tightly controlled spatial and temporal signaling. Variations in intracellular Ca^2+^ concentration modulate flagellar activity and are essential for the transition from progressive motility (PMOT) to hyperactivation, mainly through CatSper channel-mediated influx and its interaction with cAMP-dependent signaling pathways (12–14). During capacitation, Ca^2+^ exerts a concentration-dependent regulatory role, coordinating intracellular signaling pathways, protein phosphorylation and metabolic activity required for fertilization competence (15, 16). In addition, the acrosome reaction depends on a Ca^2+^-driven cascade involving both intracellular store release and extracellular influx (14).

Recent evidence in mammals indicates that Ca^2+^ homeostasis is essential for maintaining sperm function during liquid storage, as ion channels and Ca^2+^-ATPases contribute to the regulation of intracellular Ca^2+^ balance and metabolic stability. Similarly, controlled calcium regulation has been shown to preserve sperm metabolic activity and functionality during liquid storage in pig semen, highlighting the importance of calcium balance rather than absolute concentration (17).

Several studies have investigated the practical effects of calcium supplementation in semen extenders, demonstrating that controlled extracellular Ca^2+^ addition can improve sperm motility and viability, whereas unregulated Ca^2+^ influx may be detrimental. *In vitro* experiments have shown that calcium salts such as CaCl_2_ can increase progressive motility (approximately 6–16%) and viability (4–11%), while calcium ionophores or channel antagonists significantly impair sperm function, highlighting the importance of regulated Ca^2+^ entry (18, 19). In addition, extender composition has been shown to influence intracellular Ca^2+^ dynamics during cooling and thawing, with consequences on acrosome integrity and sperm survival (20). In practical applications, supplementation of extenders with CaCl_2_, in combination with metabolic modulators, has improved fertility outcomes in swine by increasing post-thaw sperm functionality and reproductive performance (21). More recent studies also confirm that Ca^2+^ homeostasis is essential for maintaining sperm metabolic activity and functional integrity during liquid storage in mammals, further supporting the relevance of calcium regulation in semen preservation (17).

In addition to Ca^2+^, increasing evidence indicates that the endogenous opioid system may modulate sperm physiology through mechanisms involving energy metabolism and motility regulation. However, the effects of opioids on sperm function are controversial, with conflicting results reported in different species, including humans and horses (22–24).

The presence of μ-, δ- and κ-opioid receptors has been documented in the spermatozoa of various species, including humans, horses, pigs, sheep and fish (24–29). These receptors are mainly distributed across the acrosomal membrane, the equatorial region, the neck and the tail. These areas are functionally relevant for motility and the acrosome reaction (25, 27, 29, 30). Activation of the μ-receptor by agonists such as morphine has been shown to reduce sperm motility, an effect that can be reversed by the antagonist naloxone (Nx) (31). Importantly, naloxone activity appears to be dose-dependent, as low concentrations may enhance motility through μ-receptor antagonism, while higher concentrations may inhibit motility via interference with δ-receptors (24). Despite these findings, the application of naloxone in semen extender formulations remains poorly characterized. Although opioid receptors have been shown to influence sperm motility in different species, including boar and equine spermatozoa (24, 29), and naloxone has been evaluated under post-thaw conditions, current evidence does not consistently support its beneficial effect on sperm preservation, suggesting that its action is species- and protocol-dependent.

Opioid receptors can interact with calcium channels and influence the entry of calcium into cells, which in turn affects ATP production and sperm motility (32, 33). This suggests a possible interaction between the opioid system and calcium metabolism in regulating sperm function. Based on this evidence, the present study aimed to evaluate the effects of calcium chloride (CaCl_2_), naloxone (Nx), and their combination on rabbit sperm quality during liquid storage, using two different commercial extenders (TRIS and Lepus), in order to assess how modulation of calcium homeostasis and opioid signaling influences sperm motility, kinematics, vitality, and morphology.

## 2 Materials and methods

### 2.1 Ethical approval

All treatments, as well as the housing and care of the animals, were carried out in accordance with EU Directive 2010/63/EU on the protection of animals used for scientific purposes. The Ethics Committee of the Department of Veterinary Medicine at the University of Bari “Aldo Moro”, Italy (protocol no. 5082 - III/13) approved the protocol and procedures.

### 2.2 Animals and breeding conditions

This study was conducted on New Zealand White male rabbits that were bred in a semi-intensive meat production facility in Poggio Imperiale, Foggia, Italy. Twenty clinically healthy breeding subjects, aged between 8 and 16 months with an average weight of approximately 4 kg, were selected. The animals underwent regular veterinary checks and were kept under uniform environmental and feeding conditions throughout the experiment to minimize variability in semen parameters between individuals.

### 2.3 Collection of seminal material and preliminary assessments

The semen was collected using a standardized procedure involving an Amantea artificial vagina and constant temperature conditions. Immediately after collection, the total volume of the ejaculate was measured using a graduated container. Sperm concentration and motility parameters were assessed using a computer-assisted sperm analysis system (AndroScope^®^, Minitube, Berlin, Germany) that was specifically calibrated for rabbits. The system was used with the standard calibration settings provided by the manufacturer, and no additional adjustments or custom configurations were applied. This provided the sperm concentration, expressed in millions of spermatozoa/ml, as well as the percentage of total and progressive motility.

The pH of each sample was measured using a digital pH meter (HI9810402HAL02, Hanna Instruments, Italy).

Sperm vitality and morphology were analyzed through differential staining with eosin and nigrosin to determine the percentage of live, dead, and morphologically abnormal cells by examining at least 200 spermatozoa per slide.

### 2.4 Preparation of the semen pool

All ejaculates were collected from each animal at a single time point and subsequently pooled to create a homogeneous sample representative of the 20 donors, following standard procedures commonly adopted in rabbit artificial insemination. A second quantitative and qualitative assessment was performed on this pool, the results of which were comparable to the average values of the individual samples. This confirmed the homogeneity of the material used for subsequent experimental tests. The entire process of collection, preliminary evaluation, and pooling was performed within a short time frame and under controlled conditions to minimize potential alterations in sperm quality.

### 2.5 Dilution and experimental treatments

The semen pool had a total volume of 13.5 ml. This was divided into two equal aliquots of approximately 6.7 ml each. One of these was intended for use with one commercial diluent and the other with a different one. The first aliquot was diluted with Lepus diluent (MEDI Chimica, Reggio Emilia, Italy), characterized by a pH between 6.9 and 7.2. This diluent contains bicarbonate (0.005% in 50 kg of raw material) and is free of calcium in any form. The second was treated with a TRIS buffer solution prepared according to the formulation described by Dal Bosco and Castellini (57). This consisted of Tris-amino-methane (3.028 g), citric acid monohydrate (1.675 g), fructose (1.250 g), and sterile distilled water to make up 100 ml. The extender was subsequently supplemented with 20% fresh egg yolk.

**Figure 1:**
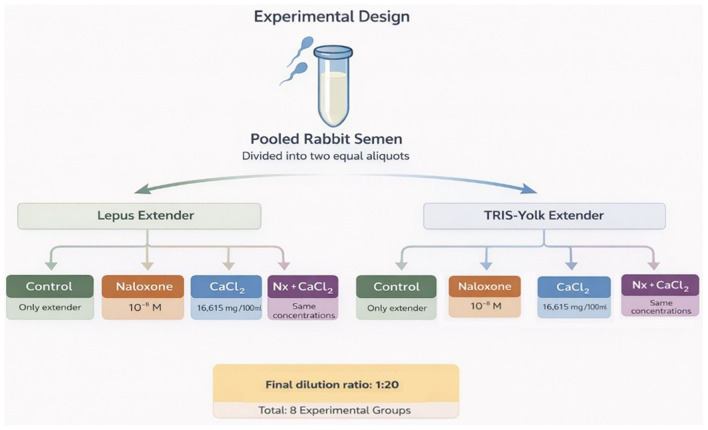
Experimental design.

Each extender sample was further divided into four treatment samples, each containing approximately 1.6 ml of semen: a control containing only the diluent; a formulation containing Naloxone (Nx) at a concentration of 10^−8^ M selected based on previous studies demonstrating beneficial effects of low-dose naloxone on sperm motility through opioid receptor antagonism (24); a formulation containing calcium chloride (CaCl_2_) at a dose of 16.615 mg per 100 ml; and a formulation containing a combination of Nx and CaCl_2_ at the same concentrations. The amount of CaCl_2_ used provided a total of approximately 6 mg of Ca^2+^ ions, corresponding to the physiological concentration present in 100 ml of rabbit ejaculate. All samples were diluted at a ratio of 1:20, resulting in a final sperm concentration of approximately 18 × 10^6^ spermatozoa/mL in each experimental group. This resulted in eight distinct experimental groups (Figure 1). For each experimental group, three analytical replicates were evaluated. For each replicate, a 2 μL aliquot of seminal material was placed on a Leja chamber (SC 10-01-04-B, Leja, GN Nieuw-Vennep, NL), pre-warmed on a laboratory heating plate, and analyzed with the aid of a Nikon Eclipse Ni phase contrast optical microscope, equipped with a heated stage, 10 × /0.25 Ph1 phase contrast objective, and computerized automatic semen analysis system (CASA) were used. All semen parameters were therefore evaluated in triplicate for each treatment combination.

### 2.6 Storage and analysis of seminal material

After collection, preliminary evaluation, and pooling, semen samples were placed in a portable temperature-controlled refrigerator to ensure controlled cooling conditions during transport (cooled at an approximate rate of −0.5 °C/min, reaching +4 °C within 2 h). Upon arrival at the Andrology Clinic laboratories at the Veterinary Hospital of the University of Bari “Aldo Moro”, samples were gently rewarmed in a water bath to 37 °C prior to analysis.

A computerized sperm analysis system [Computer Assisted Sperm Analysis (CASA)] using IVOS-Sperm CASA version 12.3 equipment (Hamilton Thorne, USA) was used to perform the kinetic analyses. Using the rabbit-specific setup (software settings: frame rate 60 Hz; minimum contrast 50; minimum data points 7; path velocity threshold 100 μm/s^−1^, average path velocity (VAP) cut-off 9.0 μm/s^−1^, medium VAP cut-off 20 μm/s^−1^; straightness threshold 70%), the following parameters were evaluated: sperm concentration (CONC, 10^6^ ml^−1^), total motility (MOT, %; >5 μm/s), progressive motility (PMOT, %; > 20 μm/s), VAP (average path velocity, μm/s), VCL (curvilinear velocity, μm/s), VSL (straight-line velocity, μm/s), DAP (average path distance, μm), DCL (curvilinear distance, μm), DSL (straight-line distance, μm), STR (straightness, VSL/VAP), LIN (linearity, VSL/VCL), ALH (amplitude of lateral head displacement), and BCF (beat cross frequency).

### 2.7 Statistical analysis

The data distribution was preliminarily verified using the Shapiro–Wilk normality test at a significance level of α = 0.05. Data were considered normally distributed since the value of p was greater than α for all the variables that were analyzed.

Differences among experimental conditions were analyzed using a two-way ANOVA in a 2 × 4 factorial design, with diluent (Lepus vs. TRIS) and treatment (control, Nx, CaCl_2_, and Nx + CaCl_2_) as fixed factors, including their interaction. When significant main effects or interactions were detected (*p* < 0.05), pairwise comparisons were performed using Tukey's honestly significant difference (HSD) *post hoc* test. The results are expressed as mean ± standard deviation (SD), and effect size was estimated using the η^2^ coefficient.

## 3 Results

The semen parameters of the 20 male rabbits included in the study were all within the normal range for the species, as shown in (Table 1).

**Table 1:** The semen parameters of the 20 male rabbits used in the study are reported below.

Rabbit	Volume (ml)	pH	Sperm × 10^6^/ml	Total motility (%)	Progressive motility (%)	Number of alive (%)	Number of dead (%)	Abnormalities (%)
1	0.6	7	324	76	63	81	14	5
2	0.58	7.5	426	64	51	72	21	7
3	1.2	7.4	253	71	63	77	17	6
4	0.9	7.3	485	65	52	71	18	11
5	0.49	7.6	368	60	54	84	14	2
6	0.55	7.4	346	62	55	81	16	3
7	0.78	7.5	397	67	56	70	20	10
8	0.94	7.5	443	69	57	87	11	2
9	1	7.6	286	72	68	81	18	1
10	0.53	7.6	361	75	65	70	20	10
11	0.74	7.7	352	61	55	71	19	10
12	0.69	7.4	423	67	58	80	16	4
13	0.48	7.6	486	68	57	83	16	1
14	0.56	7.5	376	82	73	79	17	4
15	0.62	7.6	314	81	71	83	14	3
16	0.52	7.4	349	64	54	71	21	8
17	0.55	7.5	374	73	62	88	8	4
18	0.61	7.6	339	71	63	80	14	6
19	0.53	7.5	358	62	54	70	22	8
20	0.63	7.7	367	79	63	81	15	4
Media	0.67894737	7.495	371.35	69.45	59.7	78	16.55	5.45
St. dev.	0.19977473	0.15719582	59.4875263	6.668662	6.317061275	6.01751829	3.53143776	3.18673236

The following parameters are reported: ejaculate volume (ml), pH, sperm concentration, total and progressive motility (%); Percentage of live and dead spermatozoa (%); and percentage of morphological abnormalities. The mean values and standard deviations (SD) are shown at the bottom of the table.

The semen pool obtained from the 20 breeding rabbits showed favorable semen quality parameters, as indicated by high sperm motility and vitality levels (Table 2).

**Table 2:** The semen parameters of a pool obtained from 20 male breeding rabbits are reported.

Total volume	13.5 ml
pH	7.5
Sperm concentration	368 × 10^6^/ml
Total motility	68%
Progressive motility	59%
Percentage of live/dead spermatozoa	78/16.55%
Abnormalities	5.4%

The following are reported: total volume; pH; sperm concentration; total and progressive motility; percentage of live and dead spermatozoa; and percentage of morphological abnormalities.

### 3.1 Effect of diluent and treatments on sperm quality

Two-way ANOVA showed that treatment was the main factor affecting all analyzed sperm quality parameters. In contrast, the main effect of the diluent was significant only for certain variables, while the diluent × treatment interaction was significant only for certain traits. This suggests that the effect of supplementation was consistent across extenders, but depended on the specific parameter (Table 3).

**Table 3:** Results for the different parameters expressed as the mean value in the 2 × 4 factorial arrangement [2 extenders (LEPUS and TRIS) × 4 treatments (diluent alone, diluent + Nx, Diluent + CaCl_2_; diluent + Nx + CaCl_2_)].

Parameter	Treatment group	Diluent	Diluent + Nx	Diluent + CaCl_2_	Diluent + Nx + CaCl_2_	Two-way anova results				
						**Factor**	***p*** **-value**	***F*** **-test**	η^2^	
									Value	Interpretation
MOT%	L	70.93 (5.99)a	77.07 (1.27)b	56.37 (1.02)c	84.58 (1.19)b	Group	*p* > 0.05	2.2609	0.12	Medium
						Treatments	*p* < 0.05	242.6258	0.97	Large
	T	71.2 (1.05)a	80.83 (1.72)b	57.30 (0.95)c	85.48 (0.81)b	Interaction	*p* > 0.05	0.6435	0.11	Medim
PMOT%	L	57.37 (0.76)a	73.37 (1.46)b	44.67 (1.96)c	81.77 (1.36)d	Group	*p* > 0.05	0.2539	0.16	Medium
						Treatments	*p* < 0.05	364.88	0.99	Large
	T	57.07 (4.77)a	75.53 (0.86)b	44.27 (1.96)c	82.08 (1.35)d	Interaction	*p* > 0.05	0.4523	0.07	Medium
DAP	L	24.53 (1.59)a	31.20 (0.78)b	17.23 (3.25)c	29.00 (2.23)a	Group	*p* > 0.05	3.099	0.16	Large
						Treatments	*p* < 0.05	70,653	0.93	Large
	T	32.00 (1.00)b	29.20 (1.25)b	15.87 (1.16)c	30.0 (2.51)b	Interaction	*p* < 0.05	7.4654	0.58	Large
DCL	L	40.53 (0.75)a	53.97 (1.47)b	31.2 (1.40)c	70.23 (0.91)d	Group	*p* < 0.05	9.8713	0.38	Large
						Treatments	*p* < 0.05	331.64	0.98	Large
	T	42.53 (1.40)a	58.07 (1.50)b	37.07 (2.70)ac	69.16 (4.36)d	Interaction	*p* > 0.05	2.9676	0.58	Large
DSL	L	16.47 (0.83)a	20.27 (1.19)ac	11.47 (2.14)b	21.80 (2.88)c	Group	*p* > 0.05	0.5264	0.03	Small
						Treatments	*p* < 0.05	43.90	0.89	Large
	T	15.27 (0.90)a	19.73 (0.47)ac	12.33 (0.91)b	20.80 (1.73)c	Interaction	*p* > 0.05	0.5246	0.09	Medium
VAP	L	51.47 (0.74)a	54.00 (1.49)a	29.60 (2.56)b	68.73 (0.92)d	Group	*p* < 0.05	6.0082	0.27	Large
						Treatments	*p* < 0.05	124.6478	0.96	Large
	T	54.03 (0.70)a	59.67 (0.91)ad	34.43 (5.12)c	69.17 (7.30)d	Interaction	*p* > 0.05	0.7334	0.12	Medium
VCL	L	98.23 (0.85)a	116.13 (3.73)a	61.10 (14.48)b	152.53 (8.90)c	Group	*p* > 0.05	0.0585	0.003	Negligible
						Treatments	*p* < 0.05	164.2211	0.97	Large
	T	105.90 (3.84)a	111.43 (11.75)a	54.73 (3.43)b	158.90 (2.16)c	Interaction	*p* > 0.05	1.3515	0.2	Large
VSL	L	37.27 (0.86)ab	42.80 (1.49)ac	19.07 (0.153)b	53.00 (8.86)c	Group	*p* > 0.05	3.7384	0.19	Large
						Treatments	*p* < 0.05	67.1239	0.93	Large
	T	40.00 (2.19)a	14.47 (2.25)a	26.17 (4.86)b	57.10 (3.18)c	Interaction	*p* > 0.05	1.1547	0.18	Large
STR	L	0.68 (0.02)a	0.67 (0.02)a	0.54 (0.06)b	0.72 (0.05)a	Group	*p* < 0.05	6.135	0.28	Large
						Treatments	*p* < 0.05	15.6922	0.75	Large
	T	0.68 (0.03)a	0.69 (0.01)a	0.65 (0.03)a	0.72 (0.04)a	Interaction	*p* < 0.05	3.7603	0.42	Large
LIN	L	0.37 (0.01)a	0.33 (0.01)a	0.28 (0.01)b	0.33 (0.07)a	Group	*p* > 0.05	0.0059	0.00037	Negligible
						Treatments	*p* < 0.05	8.9321	0.63	Large
	T	0.28 (0.01)b	0.35 (0.03)a	0.28 (0.01)b	0.38 (0.01)a	Interaction	*p* < 0.05	7.8623	0.60	Large
ALH	L	3.83 (0.35)a	4.03 (0.21)ab	3.13 (0.61)ac	4.97 (1.01)b	Group	*p* > 0.05	2.1576	0.16	Large
						Treatments	*p* < 0.05	16.0174	0.75	Large
	T	4.53 (0.25)b	4.59 (0.60)ab	2.90 (0.45)ac	5.20 (0.10)b	Interaction	*p* > 0.05	0.9479	0.12	Medium
BSF	L	22.47 (0.49)a	27.53 (0.70)a	19.73 (2.76)a	29.97 (2.36)b	Group	*p* < 0.05	21.2636	0.57	Large
						Treatments	*p* < 0.05	41.9133	0.88	Large
	T	31.43 (0.40)b	30.50 (1.75)a	19.93 (1.54)a	31.47 (2.58)b	Interaction	*p* < 0.05	6.868	0.56	Large
Vitality	L	0.87 (0.01)a	0.90 (0.01)a	0.77 (0.02)c	0.90 (0.01)a	Group	*p* < 0.05	160.7424	0.91	Large
						Treatments	*p* < 0.05	32.6616	0.86	Large
	T	0.94 (0.01)b	0.95 (0.01)b	0.92 (0.04)b	0.96 (0.01)b	Interaction	*p* < 0.05	11.5707	0.68	Large
Abnormalities	L	8.33 (0.58)a	8.00 (1.00)a	7.67 (1.53)a	6.00 (0.58)ac	Group	*p* < 0.05	129.94	0.89	Large
						Treatments	*p* < 0.05	14.6471	0.73	Large
	T	1.67 (0.58)b	4.33 (0.58)c	6.33 (0.8)ac	2 (0.22)b	Interaction	*p* < 0.05	10.098	0.65	Large

Statistical analysis summary for the two-way Anova reports *p*-value, *F*-test and η^2^. Different superscripts denote the statistical differences obtained by the *post-hoc* test through pairwise comparisons within each parameters. The following parameters were evaluated: total motile spermatozoa (%, MOT > 5 μm/s) and progressive motile spermatozoa (%, PMOT > 20 μm/s), velocity of average path (VAP, μm/s), velocity of curved line (VCL, μm/s), velocity of straight line (VSL, μm/s), distance of average path (DAP, μm), distance of curved line (DCL, μm), distance of straight line (DSL, μm), straightness (STR, VSL:VAP ratio), linearity (LIN, VSL:VCL ratio), ALH (lateral head displacement) and beat cross frequency (BCF). Different lowercase superscript letters within the same row indicate statistically significant differences among treatment groups (*p* < 0.05), whereas values sharing the same letter are not significantly different.

For total motility (MOT%), the main effect of treatment was highly significant (*p* < 0.05), whereas neither diluent (*p* > 0.05) nor the interaction between diluent and treatment (*p* > 0.05) reached significance. Similarly, for progressive motility (PMOT%), only treatment showed a significant effect (*p* < 0.05), while diluent (*p* > 0.05) and the interaction term (*p* > 0.05) were not significant. In both extenders, samples supplemented with naloxone, either alone or in combination with CaCl_2_, showed the highest MOT% and PMOT% values, whereas CaCl_2_ alone significantly reduced both parameters compared with the respective controls.

A similar pattern was observed for the kinetic parameters. Treatment significantly affected DAP (*p* < 0.05), DCL (*p* < 0.05), DSL (*p* < 0.05), VAP (*p* < 0.05), VCL (*p* < 0.05), and VSL (*p* < 0.05). The main effect of diluent was significant for DCL (*p* < 0.05) and VAP (*p* < 0.05), but not for DAP (*p* > 0.05), DSL (*p* > 0.05), VCL (*p* > 0.05), or VSL (*p* > 0.05). A significant diluent × treatment interaction was detected only for DAP (*p* < 0.05), whereas the interaction was not significant for DCL (*p* > 0.05), DSL (*p* > 0.05), VAP (*p* > 0.05), VCL (*p* > 0.05), or VSL (*p* > 0.05). Overall, naloxone-containing treatments were associated with higher kinetic values, whereas CaCl_2_ alone resulted in the lowest values in both extenders. In particular, the Nx + CaCl_2_ groups showed the highest values for several kinematic traits.

Regarding trajectory indices, treatment significantly affected both STR (*p* < 0.05) and LIN (*p* < 0.05). The main effect of diluent was significant for STR (*p* > 0.05), but not for LIN (*p* > 0.05). Moreover, a significant diluent × treatment interaction was found for both STR (*p* < 0.05) and LIN (*p* < 0.05), indicating that the effect of supplementation on sperm trajectory partly depended on the extender used. In general, the lowest STR and LIN values were observed in CaCl_2_-treated samples, whereas naloxone, particularly when combined with CaCl_2_, maintained values comparable to or higher than those of the corresponding controls.

For ALH, treatment had a significant effect (*p* < 0.05), whereas neither diluent (*p* > 0.05) nor the interaction term (*p* > 0.05) was significant. By contrast, BSF was significantly affected by diluent (*p* < 0.05), treatment (*p* < 0.05), and their interaction (*p* < 0.05). In general, naloxone-containing treatments, especially Nx + CaCl_2_, showed higher ALH and BSF values, whereas CaCl_2_ alone was associated with lower values.

Sperm vitality was significantly influenced by diluent (*p* < 0.05), treatment (*p* < 0.05), and their interaction (*p* < 0.05). In general, TRIS-based samples showed higher vitality values than LEPUS-based samples, and naloxone-containing treatments yielded the best overall results. The lowest vitality was observed in the LEPUS + CaCl_2_ group, whereas the highest value was recorded in the TRIS + Nx + CaCl_2_ group.

Similarly, sperm abnormalities were significantly affected by diluent, treatment, and their interaction, as shown in Table 3. Overall, TRIS-based groups showed lower abnormality rates than LEPUS-based groups, and naloxone supplementation was associated with reduced abnormalities, whereas higher values were generally observed in LEPUS-based samples and in CaCl_2_-treated groups.

Effect size analysis confirmed that treatment was the main source of explained variance for all analyzed parameters, with consistently large η^2^-values. By contrast, the contribution of diluent and of the interaction term varied according to the trait considered, ranging from negligible or small effects for some variables to large effects for DAP, STR, LIN, BSF, vitality, and abnormalities.

## 4 Discussion

This study evaluated the effects of calcium chloride (CaCl_2_), naloxone (Nx), and their combination on rabbit sperm quality during short-term storage in two different extenders, TRIS and Lepus. The two-way ANOVA confirmed that treatment was the main source of variation for all analyzed sperm quality parameters, whereas the effect of diluent was significant only for selected variables and the interaction between diluent and treatment emerged only for specific traits.

The success of artificial insemination largely depends on the use of appropriate sperm extenders, which are designed to preserve the vitality and fertilizing capacity of sperm during storage. Such extenders typically contain organic buffers such as Tris, TES or sodium citrate to stabilize the pH within the physiological range of 6.8–7.2; sugars such as glucose or fructose to provide energy; solutes to control osmotic pressure; and antibiotics to restrict bacterial growth. Numerous studies have demonstrated that adding specific additives can enhance structural stability and sperm function in various species. In rabbits in particular, the use of molecules such as heparin and buserelin has had a positive effect on semen quality (34–40).

In this context, ionic homeostasis, particularly that of Ca^2+^, plays a fundamental role in maintaining sperm function during semen storage. Several ion channels and pumps, including voltage-dependent anion channels (VDACs) and sarco/endoplasmic reticulum Ca^2+^-ATPase (SERCA), have been identified as key regulators of the balance of calcium ions inside cells, thereby contributing to the metabolic and functional stability of spermatozoa (41, 42). Studies in horses have demonstrated that adding Ca^2+^ chelating agents to freezing media can significantly enhance sperm cryotolerance (43). In pigs, Ca^2+^ has been shown to regulate intracellular ATP levels and protein phosphorylation, thereby directly influencing sperm motility (44).

The results of this study confirm that, while calcium (Ca^2+^) is essential for sperm physiology, an uncontrolled increase in its extracellular availability can be detrimental. Adding CaCl_2_ alone led to a significant decrease in motility, kinematic and vitality parameters, likely due to excessive intracellular Ca^2+^ levels. This condition is known to induce increased ATP consumption, premature capacitation and, in some cases, spontaneous acrosome reaction triggering, resulting in reduced sperm longevity and function (8–11). These findings are also consistent with recent observations in other species, such as poultry, where modulation of calcium metabolism has been shown to be crucial for maintaining fertility during semen storage (12). Another key regulator of Ca^2+^ flow is the CatSper channel, which is the primary calcium channel found in spermatozoa. First identified in mice and humans in the early 2000s (45, 46), the CatSper channel consists of four main subunits (CatSper1–4) and several accessory subunits and has since been found in numerous vertebrate species. It plays a crucial role in the processes of capacitation and hyperactivation, which are essential for spermatozoa to acquire fertilization competence (47–49).

In this complex context of ionic regulation, the endogenous opioid system acts as an additional modulator of sperm function. Asthenozoospermia, a condition characterized by impaired sperm motility, is frequently encountered in individuals with opioid dependence, suggesting an inhibitory effect of opioids on sperm motility (50). Furthermore, males with reduced sperm motility have been observed to exhibit decreased activity of aminopeptidase N, an enzyme involved in the metabolism of enkephalins. Enkephalin expression in male germ cells has also been widely documented (51, 52). These findings collectively support the hypothesis of direct involvement of the opioid system in reproductive processes.

One of the main findings of the present study was the beneficial effect of naloxone on sperm quality in both extenders. Samples supplemented with naloxone, either alone or in combination with CaCl_2_, showed the highest values for total motility, progressive motility and most kinematic parameters. The two-way ANOVA demonstrated that treatment had a significant effect on MOT% and PMOT%, whereas neither the main effect of diluent nor the interaction term was significant for these two variables. This indicates that the positive action of naloxone on the main motility traits was consistent across both extenders.

The increase in MOT% and PMOT%, together with the improvement observed in several kinetic parameters, suggests that antagonism of opioid receptors, particularly μ-receptors, may remove a physiological inhibitory mechanism affecting sperm motility. These results are in agreement with findings reported in humans, horses and pigs, where opioid receptor activation has been associated with reduced sperm motility, while naloxone administration has been shown to restore or improve movement (24, 29, 31, 52).

The localization of μ-, δ- and κ-opioid receptors in the acrosomal membrane, the neck and the flagellum provide further support for the hypothesis that the opioid system directly contributes to the controls sperm motility (25, 27). Therefore, the observed effect of naloxone may be interpreted as the result of competitive antagonism of endogenous opioids, which allows for more efficient restoration of energy processes and flagellar movement.

Another relevant findings of this study was the observed synergistic effect in groups treated with a combination of naloxone and CaCl_2_. The motility, kinematics and vitality parameters in these samples were higher than in the control group and exceeded those in the naloxone-only group. These results suggest that naloxone may modulate the entry and intracellular distribution of Ca^2+^, thereby preventing the cytosolic peaks that are responsible for the negative effects observed in samples treated with CaCl_2_ alone. Previous studies have shown that opioid receptors can interact with L-type calcium channels and intracellular signaling pathways involved in energy metabolism, such as protein kinase C (PKC) (23, 24), supporting this hypothesis. In this context, naloxone could help to maintain balanced Ca^2+^ turnover, thereby preserving ATP production and supporting flagellar activity, even when there is increased availability of extracellular Ca^2+^.

Previous studies have demonstrated that diluent composition significantly impacts sperm motility, membrane integrity, and DNA integrity (6). A comparison of the two extenders showed that the main effect of diluent was more limited than that of treatment, but remained significant for selected parameters, including DCL, VAP, STR, BSF, vitality and abnormalities. In general, TRIS-based samples showed higher vitality and lower abnormality rates than Lepus-based samples, suggesting that the composition of the extender contributed to sperm protection during storage. However, because the diluent effect was not significant for several of the major motility and kinematic variables, the present data indicate that extender composition played a secondary role compared with the supplementation. Therefore, it would be more appropriate to state that TRIS performed better than Lepus only for selected traits, rather than concluding that it was globally superior for all sperm quality parameters. The effect size analysis further supports this interpretation. Across all analyzed variables, η^2^-values associated with treatment were consistently large, indicating that treatment explained the highest proportion of variance. By contrast, the contribution of diluent and of the interaction term varied according to the trait considered, ranging from negligible or small effects for some variables to large effects for DAP, STR, LIN, BSF, vitality and abnormalities.

Previous studies have shown that naloxone activity is dose-dependent, with different effects reported at varying concentrations. However, in the present study, naloxone was used at a single concentration (10^−8^ M), and all analyses were performed on refrigerated semen samples (4 °C). The results of this study suggest that pharmacological modulation of the opioid system, through the use of naloxone at the tested concentration, may be a promising strategy for improving the quality of refrigerated semen intended for artificial insemination in rabbits. The observed synergistic effect with Ca^2+^ also indicates that optimizing the ionic and metabolic balance of spermatozoa through a combined approach could improve reproductive efficiency in intensive breeding systems.

The present findings should also be interpreted considering the storage conditions. All semen samples were refrigerated at +4 °C, and chilled storage is known to alter sperm metabolic activity, membrane fluidity and ion transport mechanisms. Cooling is known to reduce sperm metabolic rate, leading to decreased ATP production and a general slowdown of cellular processes, as demonstrated in several species including rabbits (53). This reduction in metabolic activity is associated with changes in motility and energy availability, which are closely linked to intracellular ATP levels (54). In addition, chilled storage can induce alterations in sperm membrane composition and fluidity, increasing susceptibility to cold-shock damage and oxidative stress (55, 56). These temperature-dependent changes may affect ion transport mechanisms, including calcium fluxes, and consequently influence sperm motility regulation and functional stability. Under these conditions, the effects of calcium supplementation and opioid receptor modulation may be partially dependent on the reduced metabolic and functional state of the spermatozoa. In particular, the lower metabolic demand at reduced temperatures may mitigate the detrimental effects of Ca^2+^ overload, while enhancing the regulatory role of naloxone on calcium homeostasis and energy balance. Therefore, the observed treatment effects should be interpreted in the context of refrigerated storage, which may have contributed to stabilizing sperm function and modulating the interaction between calcium dynamics and the opioid system.

This study demonstrates that naloxone significantly improves rabbit sperm quality by enhancing motility, kinematics and vitality, while reducing abnormalities. While the addition of Ca^2+^ can be detrimental if not properly modulated, combining it with naloxone helps to further enhance sperm function. These results support the hypothesis of a close functional interaction between the opioid system and calcium metabolism in spermatozoa, offering new insights into the optimisation of artificial insemination protocols in rabbits.

## 5 Conclusion

This study demonstrates that modulation of the endogenous opioid system through naloxone significantly improves rabbit semen quality during short-term storage, enhancing motility, kinematics, and vitality, while reducing sperm abnormalities. In contrast, calcium supplementation alone negatively affected sperm performance, whereas its combination with naloxone resulted in a synergistic effect, leading to the highest overall sperm quality. From an applied perspective, the addition of naloxone at the tested concentration (10^−8^ M) may represent a practical strategy to improve the quality of refrigerated semen used in artificial insemination protocols in rabbits.

However, this study has some limitations, including the *in vitro* experimental design and the absence of direct fertility outcomes. Therefore, further studies are needed to evaluate *in vivo* reproductive performance and to better elucidate the molecular mechanisms underlying the interaction between opioid receptors and calcium metabolism. Nevertheless, these results must be validated *in vivo* fertility tests, and the molecular mechanisms involved must be investigated, particularly the interaction between opioid receptors, calcium channels and mitochondrial energy metabolism. Future studies will also need to evaluate the medium- to long-term effects and those under conditions of prolonged storage.

## Data Availability

The original contributions presented in the study are included in the article/supplementary material, further inquiries can be directed to the corresponding author.
